# Supplementary material for: Identification of Freezing-Responsive microRNAs and Their Targets in Chinese Jujube by Small RNA and Degradome Sequencing
Source: Int J Mol Sci. 2026 Sep 4;27(17):7912. doi: 10.3390/ijms27177912 (PMC13566890; doi:10.3390/ijms27177912)
Supplement: Supplementary file 1 [file ijms-27-07912-s001.zip › Supplementary Material S2/novel_miR_61.pdf]

Provisional ID : novel_miR_61
Score total : 4.2
Score for star read(s) : -1.3
Score for read counts : 0
Score for mfe : 3.9
Score for randfold : 1.6
Score for cons. seed :
Total read count : 176
Mature read count : 176
Loop read count : 0
Star read count : 0

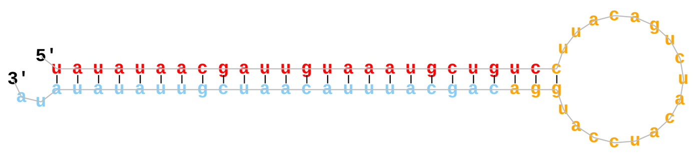


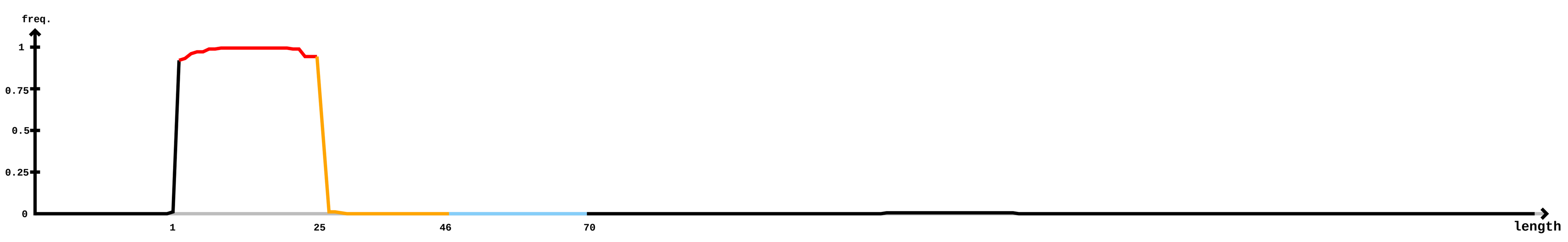


Mature Star

```
5'- aaguaucugcauaaaucaugcauauauaacgauuguaaaugcugucuuacagucuacauccauggacagcauuuacaaucguuauauauacaugauuuacgcagauacuuauauggaaaauggaguguguguauauauauauauauauauguauguauguauguauguauguuugcauguauguauuuccugcucccuuaauuucgugcaaaucuuugagcaugauucuaacauacucuuuugaaug -3'
    (((((((((.(((((((((.((((((((((((((((((((((((((................)))))))))))))))))))))))))).))))))))).))))))))))..........(((((((((((((((((((((((((....)))))))))))))))))))(((((((((.........................))))))))).................)))))))))........
```

| read | reads | mm | sample |
| --- | --- | --- | --- |
| auauauaacgauuguaaaug | 1 | 0 | LT-3 |
| auauauaacgauuguaaaugcuguc | 1 | 0 | LT-2 |
| uauauaacgauuguaaaugcu | 1 | 0 | LT-3 |
| uauauaacgauuguaaaugcu | 7 | 0 | LCK-3 |
| uauauaacgauuguaaaugcuguc | 12 | 0 | LT-3 |
| uCuauaacgauuguaaaugcuguc | 1 | 1 | LT-3 |
| uauauaacgauuguCaaugcuguc | 1 | 1 | JCK-2 |
| uauauaacgauuguaaaugcuguc | 35 | 0 | JCK-3 |
| uauauaacgauuguaaaugcuguc | 3 | 0 | LT-2 |
| uauauaacgauuguaaaugcuguc | 12 | 0 | LCK-3 |
| uauauaacgauuguaaaugcuguc | 34 | 0 | JCK-2 |
| uauauaacgauuguaaaugcuguc | 3 | 0 | JT-2 |
| uauauaacgauuguaaaugcuguc | 35 | 0 | JCK-1 |
| uauauaacgauuguaaaugcuguc | 4 | 0 | JT-3 |
| uauauaacgauuguaaaugcuguc | 13 | 0 | LT-1 |
| auauaacgauuguaaaugcuguc | 2 | 0 | JCK-2 |
| uauaacgauuguaaaugcuguc | 1 | 0 | LT-1 |
| uauaacgauuguaaaugcuguc | 1 | 0 | JCK-2 |
| uauaacgauuguaaaugcuguc | 2 | 0 | JCK-1 |
| uauaacgauuguaaaugcuguc | 1 | 0 | LT-3 |
| auaacgauuguaaaugcuguc | 2 | 0 | LT-1 |
| aacgauuguaaaugcuguc | 2 | 0 | LT-3 |
| aacgauuguaaaugcuguccuu | 1 | 0 | LT-1 |
| cgauuguaaaugcuguccAua | 1 | 1 | LT-3 |
| uauauauauauguaCguaug | 1 | 1 | JT-2 |
